# Selective Inhibition of Orexin-2 Receptors Prevents Stress-Induced ACTH Release in Mice

**DOI:** 10.3389/fnbeh.2017.00083

**Published:** 2017-05-08

**Authors:** Sujin Yun, Michelle Wennerholm, Jonathan E. Shelton, Pascal Bonaventure, Michael A. Letavic, Brock T. Shireman, Timothy W. Lovenberg, Christine Dugovic

**Affiliations:** Department of Neuroscience, Janssen Research and Development, L.L.C.San Diego, CA, USA

**Keywords:** orexin-1, orexin-2 receptor antagonist, ACTH, sleep, mice

## Abstract

Orexins peptides exert a prominent role in arousal-related processes including stress responding, by activating orexin-1 (OX1R) and orexin-2 (OX2R) receptors located widely throughout the brain. Stress or orexin administration stimulates hyperarousal, adrenocorticotropic hormone (ACTH) and corticosterone release, and selective OX1R blockade can attenuate several stress-induced behavioral and cardiovascular responses but not the hypothalamic-pituitary-adrenal (HPA) axis activation. As opposed to OX1R, OX2R are preferentially expressed in the paraventricular hypothalamic nucleus which is involved in the HPA axis regulation. In the present study, we investigated the effects of a psychological stress elicited by cage exchange (CE) on ACTH release in two murine models (genetic and pharmacological) of selective OX2R inhibition. CE-induced stress produced a significant increase in ACTH serum levels. Mice lacking the OX2R exhibited a blunted stress response. Stress-induced ACTH release was absent in mice pre-treated with the selective OX2R antagonist JNJ-42847922 (30 mg/kg po), whereas pre-treatment with the dual OX1/2R antagonist SB-649868 (30 mg/kg po) only partially attenuated the increase of ACTH. To assess whether the intrinsic and distinct sleep-promoting properties of each antagonist could account for the differential stress response, a separate group of mice implanted with electrodes for standard sleep recording were orally dosed with JNJ-42847922 or SB-649868 during the light phase. While both compounds reduced the latency to non-rapid eye movement (NREM) sleep without affecting its duration, a prevalent REM-sleep promoting effect was observed only in mice treated with the dual OX1/2R antagonist. These data indicate that in a psychological stress model, genetic or pharmacological inhibition of OX2R markedly attenuated stress-induced ACTH secretion, as a separately mediated effect from the NREM sleep induction of OX2R antagonism.

## Introduction

Orexin-A and -B are excitatory neuropeptides produced by lateral hypothalamic neurons that play important roles in the regulation of sleep/wake cycles, energy metabolism, reward-directed behavior, anxious arousal, stress responses and monoaminergic neurotransmitter release via a relatively discrete network of neuroanatomical projections (Li et al., 2014; Sakurai, 2014). The orexins stimulate two distinct G-protein coupled receptors, orexin-1 (OX1R) and orexin-2 (OX2R) that are co-located or selectively located in specific brain areas suggesting differentiated functions. The best-characterized orexinergic system involves the OX2R located on histaminergic neurons in the tuberomammillary nuclei of the hypothalamus, where these receptors play a critical role in wake promotion (Eriksson et al., 2001). The orexinergic circuits innervate most of the structures implicated in the pathophysiology of mood disorders, as well as in emotional behavior, stress responses and reward processing (Nollet and Leman, 2013; Li et al., 2014; Sakurai, 2014).

While it is well established that selective pharmacological blockade of OX2R is sufficient to promote sleep, pharmacological or genetic selective inhibition of OX1R minimally affects sleep (Dugovic et al., 2009, 2014; Mang et al., 2012; Bonaventure et al., 2015a,b; Gotter et al., 2016). In contrast, the differential contribution of OX1R and OX2R signaling in hypothalamic-pituitary-adrenal (HPA) axis regulation is unclear. Stress or orexin administration stimulates hyperarousal, adrenocorticotropic hormone (ACTH) and corticosterone release (Berridge et al., 2010) and selective OX1R blockade can attenuate several stress-induced behavioral and cardiovascular responses but not the HPA axis activation (Bonaventure et al., 2015b). As opposed to OX1R, OX2R are predominantly expressed in the paraventricular hypothalamic nucleus which is involved in the HPA axis regulation (Trivedi et al., 1998; Marcus et al., 2001).

In the present study, we investigated the effects of a psychological stress elicited by cage exchange (CE) on ACTH release in two murine models of selective OX2R inhibition, a genetic approach using mice lacking the OX2R and a pharmacological procedure using the selective OX2R antagonist JNJ-42847922 (Bonaventure et al., 2015a) and the dual OX1/2R antagonist SB-649868 (Di Fabio et al., 2011). To assess whether the intrinsic and distinct sleep-promoting properties of each antagonist could account for the potential differential stress response, separate groups of mice were implanted with electrodes for the recording of electroencephalogram (EEG) to evaluate the effects of JNJ-42847922 and SB-649868 on sleep during the light phase. An EEG sleep study was also conducted in OX2R knockout (KO) and corresponding wild-type (WT) mice under baseline conditions.

## Materials and Methods

### Animals

Studies were performed in male adult C57Bl6 mice (30–35 g; The Jackson Laboratory, Sacramento, CA, USA), and male adult C57Bl6 OX2R KO and corresponding WT mice (30–35 g; Charles River Laboratories, San Diego, CA, USA). Animals were housed individually in cages under controlled conditions with a 12:12 light/dark schedule (lights on at 6 AM) and temperature maintained at 22 ± 2°C. During the course of the studies, animals had *ad libitum* access to food and water. All animal procedures detailed in this investigation were implemented in accordance with policies established by the Guide for the Care and Use of Laboratory Animals as adopted by the United States National Institutes of Health, (NIH Publication no. 80-23 revised 1996) and the guidelines of the Institutional Animal Care and Use Committee.

### Cage Exchange Stress and Adrenocorticotropic Hormone Measurement in Mice

The CE stress procedure and ACTH measurement were performed as previously described (Bonaventure et al., 2015b). For the CE, the animal was removed from its home cage and placed into a dirty cage previously occupied by another animal for at least 1 week. As a control procedure, the animal was removed from its home cage and returned to the same cage (brief handling). Twenty minutes later, blood was withdrawn. All the experiments were conducted between 2 h and 4 h into the light phase.

On the day of the experiment, a blood sample (200 μl) was collected in a serum separator tube (Becton Dickinson, Piscataway, NJ, USA) via the submandibular punch of each mouse. Each tube was then allowed to sit on ice for 1 h and then spun at 10,000 rpm, relative centrifugal force of 9300× *g* (Eppendorf Microfuge, Hamburg, Germany) at 4°C for 10 min to allow for separation of the serum from the remaining whole-blood constituents. Serum was then collected into a sterile Eppendorf tube and frozen at −80°C until the assay for ACTH measurement was performed. The Mouse Bone Magnetic Bead Luminex Panel (EMD Millipore, Billerica, MA, USA) was used to measure serum levels of ACTH. Each sample was run in duplicate and the two values were then averaged.

Results were graphed using Graphpad Prism. Values for ACTH levels were averaged, reported as means ± SEM and expressed in pg/ml of serum. Subsequently, an outlier analysis was performed, and an outlier value was removed when the average value of duplicates was greater than 3.5 times the standard deviation from the mean. A total of 14 ACTH samples were removed as outliers (Basal ACTH: *n* = 2; ACTH in OX2 WT and KO: *n* = 2; ACTH and JNJ-42847922: *n* = 7; ACTH and SB-649868: *n* = 3).

To determine significant differences between treatment groups or genotypes, a one-way analysis of variance (ANOVA) followed by a Tukey’s multiple comparison *post hoc* test was used. For the statistical analysis of basal ACTH levels in OX2R KO and WT mice, an unpaired Student’s *t* test was used. Differences were determined to be significant if *p* < 0.05.

### Sleep Recording and Analysis

Animals were implanted with telemetric devices for polysomnographic recording of sleep-wake patterns as previously described (Dugovic et al., 2009). To determine states of vigilance, polysomnographic waveforms were acquired from two stainless steel screw electrodes that were implanted under isofluorane anesthesia in the frontal and parietal cortex for the EEG and in dorsal nuchal muscles for the electromyogram (EMG). Electrodes were coupled to a sterile two-channel telemetric device (PhysioTel F20-EET; Data Sciences International, St. Paul, MN, USA) that had been implanted in the intraperitoneal cavity. After a 2-week period of recovery from surgery, animals were transferred to their designated housing/procedure room to allow for adaptation to the recording chamber and environment.

EEG and EMG signals were recorded for up to 12 h post drug administration and were digitized at a sampling rate of 100 Hz on an IBM PC-compatible computer using Dataquest A.R.T software (Data Sciences International, St. Paul, MN, USA). Using the computer software program SleepSign (Kissei Comtec, Nagano, Japan), consecutive EEG/EMG recordings were divided into individual 10-s epochs that were then visually assigned vigilance states based upon conventional criteria for wake, non-rapid eye movement (NREM) sleep REM sleep as described previously. Analysis of sleep-wake parameters included latency (onset) to NREM sleep (defined as the time interval to the first six consecutive NREM epochs) and REM sleep (the first two consecutive REM epochs post injection), and the duration of wake, NREM and REM sleep.

Results were averaged and expressed as mean ± SEM in defined time intervals. To determine whether differences were significant at a given interval, either a paired Student’s *t* test for NREM and REM sleep latencies, or a two-way repeated measures ANOVA (interaction Time × Treatment) followed by a Bonferroni *post hoc* test for NREM and REM sleep durations was performed.

### Drugs

JNJ-42847922, a selective OX2R antagonist (Bonaventure et al., 2015a), and SB-649868, a dual OX1/2R antagonist with similar potency at both receptor subtypes (Di Fabio et al., 2011), were synthesized at Janssen Research and Development, L.L.C. Both compounds were orally administered at the dose of 30 mg/kg in a volume of 10 ml/kg. JNJ-42847922 was formulated in 30% sulfobutylether-beta-cyclodextrin and SB-649868 was suspended in 0.5% methylcellulose.

## Results

### Inhibition of OX2R on Cage Exchange Stress-Induced ACTH Release

All the experiments were conducted between 2 h and 4 h into the light phase.

**Figure 1:**
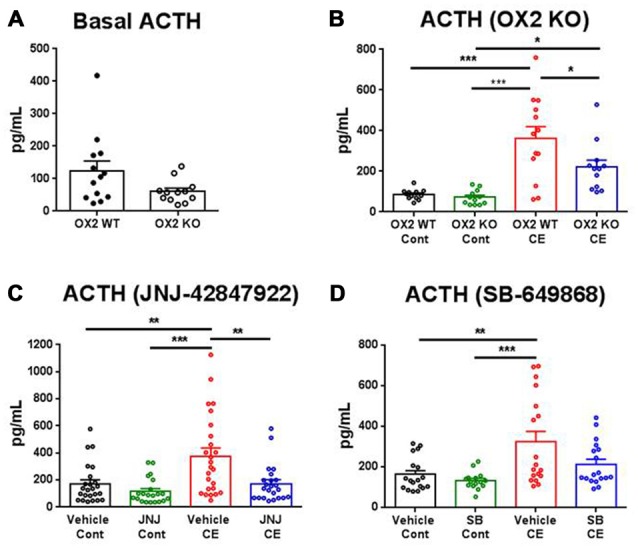
**Effects of orexin-2 (OX2R) inhibition on cage exchange (CE) stress-induced adrenocorticotropic hormone (ACTH) release in mice**. ACTH levels were measured in non-treated OX2R knockout (KO; *n* = 12) and wild-type (WT; *n* = 13) mice under baseline **(A)** and CE or control (Cont) conditions **(B)**, and in treated mice orally dosed 30 min prior to CE or Cont procedure with the OX2R antagonist JNJ-42847922 (30 mg/kg) or vehicle (*n* = 21–25 animals per condition) **(C)** and with the dual OX1/2R antagonist SB-649868 (30 mg/kg) or vehicle (*n* = 15–18 animals per condition) **(D)**. Individual values of ACTH and means (± SEM) are expressed in pg/ml of serum. Statistical significance (*p < 0.05, **p < 0.01 and ***p < 0.001) was based on one-way analysis of variance (ANOVA) followed by a Tukey’s multiple comparison *post hoc* test.

In non-treated OX2R KO and WT mice, serum ACTH levels were evaluated under baseline conditions and in a separate experiment, 20 min following the CE or the control handling procedure. Basal ACTH levels were not significantly different between the two genotypes (Figure 1A). For the stress experiment, there was a significant main effect (four treatment conditions; *F*_(3,45)_ = 14.88, *p* < 0.001). CE-induced stress produced a significant increase in ACTH serum levels in WT (three-fold increase, *p* < 0.001) whereas mice lacking the OX2R exhibited a blunted stress response (two-fold increase, *p* < 0.05) relative to their respective control conditions under which ACTH levels were very similar to each other (Figure 1B).

In pharmacologically treated mice, the effects of the selective OX2R antagonist JNJ-42847922 (30 mg/kg) and the dual OX1/2R antagonist SB-649868 (30 mg/kg) on ACTH levels were evaluated. Animals were orally dosed with the compound or its vehicle 30 min prior to CE or control handling, and blood was drawn 20 min later. The analysis of ACTH levels in mice receiving JNJ-42847922 or vehicle revealed a significant main effect (four treatment conditions in separate groups; *F*_(3,89)_ = 8.82, *p* < 0.001). In control conditions, JNJ-42847922 had no effect on ACTH serum levels as compared to the values obtained after vehicle treatment (Figure 1C). In vehicle treated mice, CE-induced stress produced a significant increase in ACTH levels as compared to the control condition (+119%, *p* < 0.01). In contrast, no significant changes in ACTH levels were found between groups of mice treated with JNJ-42847922 in CE and in control conditions. Thus, stress-induced ACTH release was virtually absent in mice pre-treated with the selective OX2R antagonist JNJ-42847922.

In mice receiving SB-649868 or vehicle, there was a significant main effect (four treatment conditions in separate groups; *F*_(3,65)_ = 6.77, *p* < 0.001). Similarly, to the results obtained with JNJ-42847922 in control conditions, SB-649868 had no effect on ACTH levels as compared to the values obtained after vehicle treatment. CE-induced stress produced a significant increase in ACTH levels as compared to the control condition in mice treated with vehicle (+98%, *p* < 0.01). However, pre-treatment with the dual OX1/2R antagonist SB-649868 only partially attenuated the increase of ACTH, as no significant difference was found between mice pretreated with vehicle and with SB-649868 under CE stress conditions (Figure 1D).

It is worth mentioning that in response to CE, ACTH release induced by the stressor showed a large variability between animals in all groups of mice, not only in OX2R KO and WT (from Charles River Laboratories, Wilmington, MA, USA; Figure 1B) but also in the C57Bl6 mice (from The Jackson Laboratory) used for both pharmacological tests (Figures 1C,D). It is possible that a number of animals seem to be vulnerable whereas others seem resilient to the stress. However, differences between genotypes or drug effects would have been difficult to evaluate by taking into account the vulnerability to stress. In pharmacologically treated mice, all the experimental conditions were identical except for the vehicle (30% sulfobutylether-beta-cyclodextrin in solution for JNJ-42847922 and 0.5% methylcellulose in suspension for SB-649868) which may have contributed to the difference in variability between the experiments with the two compounds. This factor more likely minimally affected the results since the standard deviation was consistently higher across all four conditions in the experiment with JNJ-42847922.

### Inhibition of OX2R on EEG Sleep

To assess whether the intrinsic and distinct sleep-promoting properties of each antagonist could account for the potential differential stress response, a separate group of mice (*n* = 7) implanted with electrodes for EEG sleep recording were orally dosed with JNJ-42847922 (30 mg/kg) or vehicle, then with SB-649868 (30 mg/kg) or vehicle. Experiments were conducted in a randomized cross over design, and a minimum of 3 days washout period were allowed between two treatments. The sleep test was started at 2 h into the light phase (mice were dosed at 8:00 am) and EEG/EMG was recorded up to 12 h following the dosing. Therefore, the first 2 h of sleep analysis corresponds to the same time interval when the stress experiment was conducted.

**Figure 2:**
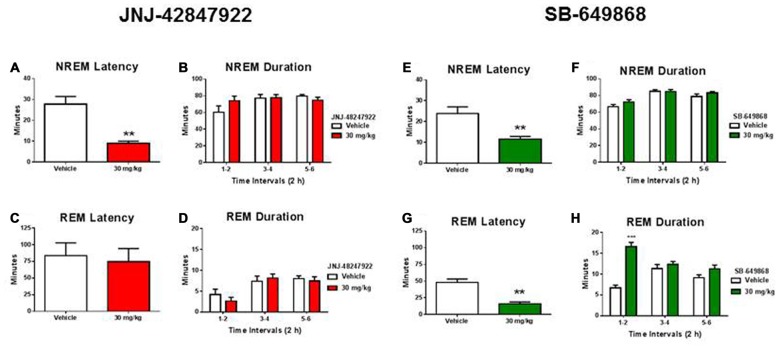
**Differential effects of OX2R and dual OX1/2R antagonism on non-rapid eye movement (NREM) and REM sleep in mice**. NREM sleep latencies **(A,E)** and durations **(B,F)** and REM sleep latencies **(C,G)** and durations **(D,H)** after oral dosing with the OX2R antagonist JNJ-42847922 (30 mg/kg) or vehicle (*n* = 7 animals per condition; left panel) and with the dual OX1/2R antagonist SB-649868 (30 mg/kg) or vehicle (*n* = 7 animals per condition; right panel) are represented as means (± SEM) and are expressed in minutes. **p < 0.01 and ***p < 0.001 vs. vehicle, based on a paired Student’s *t* test for NREM and REM sleep latencies or a two-way repeated measures ANOVA (interaction Time × Treatment) followed by a Bonferroni *post hoc* test for NREM and REM sleep durations.

As compared to their respective vehicles, both compounds significantly reduced the latency to NREM sleep (Figures 2A,E) without producing a significant impact on NREM sleep duration (Figures 2B,F). In contrast, a significantly prevalent REM-sleep promoting effect was observed in mice treated with the dual OX1/2R antagonist (Figures 2G,H) but not in mice treated with the selective OX2R antagonist (Figures 2C,D). As compared to vehicle treatment, SB-649868 administration produced a marked reduction in REM sleep latency (Figure 2G), and the time course analysis revealed a significant effect (interaction Time × Treatment; *F*_(2,24)_ = 17.84, *p* < 0.001) with a two fold increase in REM sleep duration in the first 2 h (Figure 2H).

Following the same line of reasoning, basal sleep states were evaluated in two groups of OX2R KO and corresponding WT mice during the light phase. The time spent in both NREM and REM sleep determined per 2 h-intervals for the 12 h-light phase did not differ significantly between OX2R KO and WT mice (Table 1).

**Table 1:** **Duration of non-rapid eye movement (NREM) and REM sleep during the 12-h light phase in OX2R WT and OX2R KO mice under baseline conditions**.

	NREM Duration		REM Duration	
	OX2R WT	OX2R KO	OX2R WT	OX2R KO
1–2 h	63.1 ± 4.6	58.7 ± 3.4	6.5 ± 1.0	5.8 ± 1.3
3–4 h	72.5 ± 3.6	72.3 ± 4.2	8.7 ± 1.0	7.5 ± 0.8
5–6 h	75.0 ± 2.4	74.6 ± 2.2	11.6 ± 1.2	9.5 ± 1.0
7–8 h	78.4 ± 2.9	73.8 ± 2.4	10.6 ± 1.2	8.9 ± 0.7
9–10 h	72.4 ± 3.2	69.3 ± 2.5	9.4 ± 1.1	8.6 ± 0.5
11–12 h	69.9 ± 3.5	67.5 ± 1.4	8.6 ± 1.1	8.6 ± 0.6

*Mean (± SEM) values (n = 10 OX2R WT, n = 10 OX2R KO) are determined per 2-h intervals and are expressed in minutes*.

## Discussion

The present study demonstrated that in a model of psychological stress, increased ACTH secretion can be prevented by a selective OX2R antagonist and significantly reduced in mice lacking the OX2R.

We confirmed that the CE procedure is a suitable stress model to reveal an activation of the HPA axis. Literature data indicate that psychological stress induced by CE produced an increase in body temperature, activity, arterial pressure and heart rate in mice (Oka et al., 2003; Lee et al., 2004) and acute insomnia in rats (Cano et al., 2008). The present results obtained with the CE model complement a previous study where we demonstrated that selective OX1R blockade did not affect the CE stress-induced ACTH release in mice (Bonaventure et al., 2015b). In a previous investigation, pre-treatment with the selective OX1R antagonist GSK-1059865 did not prevent yohimbine-induced plasma corticosterone release in rats (Gozzi et al., 2013). In contrast, increase of ACTH levels elicited by swimming stress in rats were attenuated with prior central infusion of a putative selective OX2R antagonist (Chang et al., 2007). Altogether, these data indicate a specific OX2R-mediated effect which is consistent with the predominant expression of OX2R vs. OX1R in the paraventricular hypothalamic nucleus (Trivedi et al., 1998; Marcus et al., 2001).

Interestingly, we found that pre-treatment with the dual OX1/2R antagonist SB-649868 only partially attenuated the increase of ACTH. It has been reported that administration of the dual OX1/2R antagonist almorexant in rats did not alter the release of corticosterone in basal and various stress conditions (novelty, social stress or restraint stress) and ACTH release after corticotropin-releasing factor challenge (Steiner et al., 2013). Furthermore, pretreatment with SB-649868 had no impact on increases in pulse rate, plasma cortisol and ACTH in the insulin tolerance test model in humans (Patel et al., 2014). It seems unlikely that SB-649868 would act as a preferential OX2R antagonist since the compound displays similar potency at both receptor subtypes (Di Fabio et al., 2011).

In rat stress models assessing cardiovascular changes, almorexant has been shown to reduce the pressor and tachycardic responses evoked by novelty (animal placed in a clean box with no bedding) and conditioned fear (Furlong et al., 2009). Significantly, simultaneous blockade of OX1R and OX2R produced a stronger effect than the blockade of either receptor alone in reducing the cardiovascular and locomotor responses of novelty stress, suggesting synergistic effects (Beig et al., 2015). In the CO_2_-induced panic model, the pressor and bradycardic responses evoked by hypercapnia could be attenuated by selective OX1R but not OX2R blockade, and unexpectedly not affected by dual OX1/2R antagonism (Johnson et al., 2015).

The results obtained in the present study indicate that additional OX1R blockade might diminish the efficacy of OX2R antagonism in suppressing the stress-induced HPA axis activation. This suggestion could be validated by testing the effects of co-administration of a selective OX1R antagonist with a selective OX2R antagonist. Numerous studies have demonstrated a key role for orexin signaling in the regulation of stress responses and suggested that the respective contribution of OX1R and OX2R might depend on the type of stressor. While stress-induced HPA axis activation seems to specifically engage OX2R, the functional interaction between the two receptor subtypes on neuroendocrine response needs to be further investigated.

The present EEG sleep study showed that when administered at the beginning of the light phase in mice, the OX2R antagonist JNJ-42847922 and the dual OX1/2R antagonist SB-649868 similarly reduced the latency to NREM sleep without significantly affecting its duration. Although a marked REM sleep-promoting effect was observed in mice treated with the dual OX1/2R antagonist, REM sleep latency and duration was not affected in mice receiving the selective OX2R antagonist. We have previously reported that JNJ-42847922 specifically promoted NREM sleep in rats during the light phase and in mice during the dark phase and that REM sleep was not altered in both species (Bonaventure et al., 2015a). In addition, we found that spontaneous sleep during the entire light phase was not altered in OX2 KO as compared to WT mice, whereas OX2R KO mice spent more time in NREM sleep during the dark phase only (Bonaventure et al., 2015a). The dual OX1/2R antagonist SB-649868 has been shown to promote predominantly REM sleep over NREM sleep in rats when administered either during the light or dark phase (Di Fabio et al., 2011; Dugovic et al., 2014), but its effects on sleep in mice have not been reported to date. Increases in both NREM and REM sleep duration have been observed in mice treated with other dual OX1/2R antagonists, almorexant and suvorexant, although these compounds were tested during the dark phase only (Mang et al., 2012; Gotter et al., 2016).

Taken together, the present results demonstrated that the efficacy of selective OX2R inhibition on stress-induced ACTH release are separate from the sleep effects of OX2R antagonism. Therefore, the suppression of ACTH secretion elicited by the CE stress was not due to the NREM sleep-inducing effects of OX2R inhibition. On the other hand, a speculative hypothesis is that the primarily REM sleep promotion produced by dual OX1/2R antagonists in animal studies might prevent a complete suppression of stress-induced ACTH release. Association between excessive increase in HPA axis activity and REM sleep disinhibition in major depression is well acknowledged (Steiger, 2002; Antonijevic, 2008). For instance, an inverse correlation between nocturnal cortisol concentrations and REM sleep latency has been shown in depressed patients (Poland et al., 1992) and under stressful conditions in healthy volunteers (Gemignani et al., 2014). In an animal model of depression, exposure to prenatal stress in rats resulted in long-term increase in REM sleep duration which was positively correlated to basal or stress-induced corticosterone levels (Dugovic et al., 1999).

In conclusion, in a psychological stress model, genetic or pharmacological inhibition of OX2R markedly attenuated stress-induced ACTH secretion, as a separately mediated effect from the NREM sleep induction of OX2R antagonism. Dual OX1/2R and selective OX2R antagonists have been developed as therapeutics for insomnia, and clinical efficacy has been demonstrated for the dual OX1/2R antagonist SB-649868 (Bettica et al., 2012) and the selective OX2R antagonist JNJ-42847922 (Bonaventure et al., 2015a). Based on these new preclinical data showing that a selective OX2R antagonist prevented stress-induced ACTH secretion, we propose that a selective OX2R antagonist might be suitable for the treatment of hyperarousal insomnia characterized by an over activity of the HPA axis. Because of the desensitization of the HPA response after repeated stress exposure, only the effects of an acute stress have been investigated in most of the animal models including the present model, making it difficult to translate into the clinic. However, an innovative study (published during the review process of the manuscript) confirmed the involvement of OX2R in acute stress response, but more importantly demonstrated that the OX2R antagonist MK-1064 could also reduce the HPA response to repeated stress under conditions of high orexin release using a chemogenetic rat model of orexin activation (Grafe et al., 2017).

## Author Contributions

SY conducted research, analyzed the data and contributed to manuscript writing; MW and JES analyzed the data; PB participated in the research design and contributed to manuscript writing; BTS and MAL provided compounds; TWL participated in research design; CD designed research, analyzed the data and wrote the manuscript.

## Conflict of Interest Statement

The authors have reviewed the journal’s policy and have the following competing interests all the authors are full-time employees of Janssen Research and Development, L.L.C. This does not alter the authors’ adherence to the journal’s policies on sharing data and materials.
